# Nepotistic Hiring and Poverty From Cultural, Social Class, and Situational Perspectives

**DOI:** 10.3389/fpsyg.2022.780629

**Published:** 2022-03-22

**Authors:** Luke Jain, Éva Gál, Gábor Orosz

**Affiliations:** ^1^Massachusetts Institute of Technology, Cambridge, MA, United States; ^2^Doctoral School of Evidence-Based Assessment and Psychological Interventions, Babes Bolyai University, Cluj-Napoca, Romania; ^3^Université d’Artois, Unité de Recherche Pluridisciplinaire Sport Santé Société, Liévin, France

**Keywords:** ethical decision making, hiring, poverty, United States, Hungary, nepotism

## Abstract

Being poor can influence how one makes ethical decisions in various fields. Nepotism is one such area, emerging as kinship-based favoritism in the job market. People can be poor on at least three levels: one can live in a poor country (cross-cultural poverty), be poor compared to others around them (socio-economic poverty), or feel poor in their given situation (situational poverty). We assumed that these levels can simultaneously influence nepotistic hiring decisions among Hungarian (*N* = 191) and US participants (*N* = 176). Prior cross-cultural, non-experimental studies demonstrated that nepotism is more prevalent in poorer countries such as Hungary than in richer countries such as the United States. However, contrary to our expectations, in our non-representative, preliminary study, US participants showed stronger nepotistic behavioral tendencies than Hungarians (cross-cultural level). Furthermore, people with lower socioeconomic status had less nepotistic intentions than richer people (socio-economic level). When participants were asked to imagine themselves as a poor person (situational level), they tended to be more nepotistic than had they imagined themselves to be rich. Finally, nepotistic hiring intentions were in general stronger than non-nepotistic hiring intentions. These seemingly paradoxical results were interpreted in the light of the COVID-19 job market context and were explained by the mechanisms described by research on wealth and immoral behaviors, as well as the presence of risk aversion.

## Introduction

Being poor can be devastating, creating various moral dilemma situations that one would not encounter if they were wealthy. These scenarios have existed for a long time, such as Kohlberg’s (1973) now-famous Heinz Dilemma. In it, a poor man called Heinz steals a drug to cure his sick wife. In this situation, there would not be a realistic ethical dilemma if Heinz was rich and could afford the cure. Clearly, socioeconomic status plays a major role in such moral dilemmas, and this has only become more relevant due to the COVID-19 situation.

Poverty is not only a multifaceted phenomenon, but also a multilevel construct. First, one can live in a less affluent country where resources such as well-paid and stable positions are scarce, leading nepotism to have huge, long-lasting benefits. Second, one can be poor compared to other people in their society. Large hierarchical distances can give those at the top a sense of entitlement and safety to act nepotistically, meaning the rich feel safe helping their relatives in nepotistic ways more frequently than their poor counterparts. Third, one can find themselves in a situation when they become poor and vulnerable as the result of a sudden threatening situation. For example, an experimental situation in which people imagine that they are poor can create such framing. These situations can be dangerous, and people may become risk-seeking to avoid further negative consequences. These threatening situations can also motivate people to give a job to a loved one for the sake of familial stability. The present work is among the first tentative trials to examine these levels of poverty in the field of nepotistic decision making.

### Nepotism

Nepotism is kinship-based favoritism in the job market. People everywhere utilize their social connections to gain employment (Granovetter, 1973), but how they use them depends on the relationship between the employer and potential employee (Akcinar, 2015). Nepotism contrasts meritocracy, as a relative is not always as suited for a job as a different non-relative candidate. However, there are advantages to securing jobs through nepotism. It can provide financial security to one’s family or create a friendly, trusting work environment (Hooker, 2009). This has been seen around the world. For instance, in post-Soviet Ukraine, non-monetized close connections or friendships (*blat*) are common ways for graduates to find jobs (Onoshchenko and Williams, 2013). Therefore, such trust-building strategies lead to nepotistic decisions (Nie and Lämsä, 2015).

Family businesses provide 40–70% of the world GDP (Zellweger, 2017). In Hungary, half of the businesses are family business (Csákné Filep, 2012), and in the US, approximately 62% of employees are employed in family owned businesses (Astrachan and Shanker, 2003). Within these companies, the paternal head of the family frequently leads the business in some manner, especially regarding hiring (Gersick et al., 1999). For them, choosing to hire a loved one vs. a stranger can be not only an ethical dilemma, but a business management question as well. We assume that such business management questions are treated differently in a resource-rich vs. a resource-poor environment. In the following, we will describe potential mechanisms through which poverty can create situations in which nepotism may appear. This line of thought will cover the following three levels: cross-cultural differences, socioeconomic differences, and situational threats.

### Cross-Cultural Level of Poverty

The World Economic Forum (Van de Vliert, 2011) studied cross-country variation of perceived nepotism in 118 countries. The United States was ranked lowest while Hungary was ranked 31st. Van de Vliert’s measure appears to be very stable over time, so the rankings from the 2006 to 2008 dataset may still be relevant in 2020. In Hungary, hiring a family member means trusting that this new employee will perform better than an “external” employee (Bogáth, 2016). In the US, instead of using the recommendations of strong links like close friends or family members, using weak links is more accepted (Granovetter, 1973). Furthermore, using nepotism to get a job has a negative connotation and it casts as “undue preference” for one’s in-group (Bellow, 2004).

Similar to nepotism (Van de Vliert, 2011), collaborative dishonesties such as corruption (Aidt, 2009) or collaborative cheating on exams (Orosz et al., 2018) are inversely related to a nation’s wealth. While corrupt acts might help facilitate deals (e.g., Huntington, 1968), collaborative forms of cheating can aid the cheaters in the long run (Poltorak, 1995), and family members can create high profits for their family business, all of these can also be inefficient and may contribute to more poverty from the perspective of broader communities (Mustapha, 2014).

We suppose that a country’s wealth (i.e., US and Hungary) can serve as a basis for judgment regardless of the individual’s position within the social hierarchy or other situational factors. Our hypothesis connects these problems to the national level: people from less wealthy countries will be willing to engage in nepotism more to protect their loved ones in a harsh environment.

### Socioeconomic Level of Poverty

At the social class level, the underlying status of a given individual may influence the social or internalized norms (Grolleau et al., 2016) that guide their moral judgment. We believe that in reference to this status, truly rich people will engage in nepotism more. Although poor people have less resources (Oakes and Rossi, 2003), they are willing to be more prosocial with what they have (Piff et al., 2010), since they are embedded in social networks where mutual relationships and support are essential (Lamont, 2000; Kraus and Keltner, 2009). Thus, these individuals tend to be external or other-oriented (Piff et al., 2016) and are more sensitive to the needs of others (Kraus and Keltner, 2009).

The converse also holds true: people of higher socioeconomic status tend to be less prosocial. One way this tendency might manifest itself is through illegal behaviors like nepotism. Individuals with higher SES are more likely to break the law, cheat, or engage in other unethical behaviors in the workplace (e.g., receiving bribes) due to their favorable attitudes toward greed (Piff et al., 2012), greater resources, independence (Kraus et al., 2012), and self-focused social cognition that emphasizes personal goals and motivations (Kraus et al., 2011, 2012). They usually exhibit higher narcissistic tendencies and entitlement (Piff, 2014), spend their income on self-interested goods (Frank, 1999), donate less (Independent Sector, 2002), and behave more selfishly (Piff et al., 2010). Thus, we hypothesized that more affluent participants would be more willing to be nepotistic.

### Situational Level of Poverty

We hypothesized that at the situational level, the propensity to cheat varies with the severity of the perceived financial threat of a given situation. We predict that the magnitude of this threat relies upon the in-scenario imagined social status participants are framed in. If a participant takes the position of a poor character (like Heinz) in an imaginary scenario, he or she will be more willing to engage in nepotism. The opposite will be true for subjects that take the position of rich character, therefore engaging in the behavior less.

Normally, people avoid taking risks, choosing outcomes that are certain rather than ones that are “possible” (Kahneman and Tversky, 2013). Contrarily, individuals of lower SES are more willing to take financial risks (Kahneman and Tversky, 1979; Brieant et al., 2021). This falls under the umbrella of prospect theory, which tells that people will engage in risky behaviors when threatened with high-probability losses (Bosch-Domènech and Silvestre, 2010). For them, some dangerous decisions are necessary for survival (Trimpop, 1994), as a large portion of their income is devoted to survival necessities like food or housing (Banerjee and Duflo, 2007). Furthermore, something like losing a loved one is absolutely devastating to anyone (Shear, 2012), and for the sake of those people, an individual can more easily justify self-sacrifice (Heinemann, 2014) or illegal behaviors. Thereby, as our participants imagine themselves in a lower socioeconomic class, they will be thrust into these loss situations and forced to avoid the threatening risks.

### The Present Study

In summary, we have a hypothesis for each level of poverty: If a participant is from a less affluent country (i.e., United States and Hungary), has high socioeconomic status, or takes the position of a poor person in a dilemma situation, they will manifest more nepotistic hiring intents.

## Methods

### Procedure and Materials

Participants were requested to voluntarily complete an anonymous online survey with demographic questions and a moral dilemma story for approximately 10 min. As the present one was a preliminary vignette study without any sort of data gathering funding, we advertised the experimental link on social media platforms. Participants did not receive any sort of compensation. We based our sample size decision on Akcinar’s (2015) studies who used similar methods to compare US and Turkish respondents. They were informed of data collection procedures and any associated risks with the information. They would first answer questions related to the dilemma and then fill out details about themselves. The dilemma had four different versions based upon two variables (situational framing: rich vs. poor and nepotistic vs. non-nepotistic hiring decision). Finally, participants were debriefed.

#### Moral Dilemma

Each version of the dilemma was a handwritten vignette based upon a nepotism situation within the world of the pandemic developed after rigorous pretesting. As it can be seen in the textbox below, they were designed to be culturally understood in Hungary and the US. In order to reduce the influence of social desirability bias, participants were not forced to choose between making a nepotistic or non-nepotistic decision. Instead they were asked to rate the likelihood they would act in a similar manner as the person in the vignette who acted in a nepotistic/non-nepotistic manner.

Participants were randomly allocated to one of the manipulated conditions in a 2 × 2 experimental design: situational poor vs. rich imagined situations as well as non-nepotistic vs. nepotistic decisions (see Table 1). Furthermore, we used as a measured (not manipulated) predictor the SES of the participants and the culture. As the Hungarian and the US samples were not similar in many sociodemographic variables, we ran additional analyses to control for them. The data gathering occurred on qualtrics and we used R to analyze the data.

**TABLE 1:** Dilemma material along the conditions.

Intro of the dilemma: “Bob is a supervisor at 7/11” “/*and a responsible father of 3 children*. He had to recently lay off a worker. The employee was on probation and started showing COVID-19 symptoms, which made his productivity much lower, leading to a big drop in sales. Bob fires him, but in order to protect his*/his family’s* income, he needs to find someone to fill the opening as soon as possible.	
Situational Poor Version: Bob is poor and has used no money for advertisements for his shop, leading to his store becoming very unknown and unpopular. Bob*/’s family* cannot go a month without getting paid because they are behind on their rent payment, and don’t want their debt to build up to the point that they could become homeless. These worries” “*/about his loved ones* are constantly on Bob’s mind.	Situational Rich Version: Bob is wealthy and has used his money for countless advertisements for his shop, leading to his store becoming very well-known and popular. Business is booming and he is making a huge amount of money nowadays. If Bob wants to maintain this high income for his*/his family and children’s future*, he needs to replace the unproductive worker immediately so that his store keeps its reputation.

Employment decision: At the same time, someone*/his oldest son* lost his job to the pandemic, so Bob hires him. Although, he realizes very quickly that the worker*/his son* is much slower and less qualified than the previous employee. Despite this, he keeps the worker/*his son* on the staff”.	

*The text of the experimental material was altered in each condition which are marked with underlined (non-nepotistic) and italicized (nepotistic) characters. The alterations for the situation-based rich vs. poor cases are denoted by separate paragraphs. We had a between subject design, only one of the situational conditions appeared for participants to read.*

### Participants

In the Hungarian sample, we received responses from 191 persons (62% female, 16.5% male, 21.5% did not report gender, aged between 18 and 51, *M_*age*_* = 23.91; *SD_*age*_* = 6.51). Regarding the highest level of education, 44.5% reported that they had a high school degree, 33.5% had a higher education degree (20.9% missing). Among them, 44% reported that they belong to a minority group (e.g., people with a Transylvanian identity currently living in Hungary or belonging to a gypsy community) and 35.1% reported that they do not belong to a minority group (20.9% missing). Using the MacArthur SES ladder, participants reported a mean SES of 6.27 with *SD* = 1.34 (ranged from 3 to 10). The theoretical range of the ladder’s scores is 1–10.

In the United States, we received responses from 176 persons (40.9% female, 57.6% male, 2.3% did not report gender, aged between 15 and 85 years, *M_*age*_* = 33.77; *SD_*age*_* = 12.88, 4% did not report age). Regarding the highest level of education 10.8% reported having no degree at all, 17.6% reported that they had a high school degree, and 69.3% had a higher education degree (2.3% missing). On the basis of ethnicity, 64.2% reported that they were Caucasian, 5.7% reported Hispanic or Latino, 9.1% reported African American, 2.8% reported Native American, 12.5% reported Asian or Pacific Islander, and 2.3% reported “Other” (3.4% missing). Using the MacArthur SES ladder, participants reported a mean SES of 6.44 with *SD* = 2.03 (ranged from 1 to 10).

The two samples were not different regarding their MacArthur ladder (*t* = −0.912, *p* = 0.319). However, there were more female participants in the Hungarian sample [χ^2^(1, *N* = 320) = 47.18, *p* < 0.001] and there were more US participants with higher education degrees [χ^2^(1, *N* = 323) = 34.27, *p* < 0.001]. Hungarians were also significantly younger [*t*(318) = −8.49, *p* < 0.001]. In the US group, there were less people who reported that they belong to a minority group than in Hungary [χ^2^(1, *N* = 321) = 15.86, *p* < 0.001].

### Measures

#### Nepotistic Behavioral Intention Measure

One item was rated on a seven-point Likert scale (1 = Extremely unlikely to 7 = Extremely likely): “If you were in Bob’s situation, how likely are you to do the same?”

#### Character Evaluation Measures

Participants were asked to rate how considerate and sympathetic the character was a) in general, b) from the perspective of their family, and c) from the perspective of his broader community. The items were rated on a five-point Likert scale from 1 (“Not at all”) to 5 (“Very much). According to the principal component analysis, the six items loaded on one factor (standardized loadings ranged between 0.63 and 0.84) with internal reliability (α = 0.86).

#### Socioeconomic Status

We employed the MacArthur Scale of Subjective Social Status in order to determine each participant’s real socioeconomic status. The scale gives participants a picture of a 10-rung ladder. They were told that the richest people were at the top and the poorest at the bottom, and instructed to place an X where they would be relative to their financial status (Goodman et al., 2001). We also asked for their level of education. In the case of the Hungarian sample, the ladder was a better indicator of SES since the participants were all taken from a university. In the United States, though, both methods worked effectively, and the ladder was perhaps even more applicable, since a wider breadth of educational demographics was assessed.

### Analytic Strategy

With OLS regression analyses, in an imaginary situation, we examined the effect of four main predictors (independent variables): two experimentally manipulated, and two measured ones. Nepotistic and non-nepotistic condition as well as situational imagined poverty were experimentally manipulated. However, SES and culture was not manipulated. The most important outcome (dependent) variables were *behavioral intentions* to behave the same way as the person in the scenario and the *evaluation* of this person. For the sake of simplicity, we will show the standardized results. In additional analyses, we also controlled for differences in demographic variables (gender, age, ethnicity, and level of education).

## Results

### Nepotistic Behavioral Intentions in the United States and in Hungary

U.S. participants reported stronger nepotistic behavioral intentions (*M* = 5.00, *SE* = 0.13) than Hungarian participants (*M* = 3.53, *SE* = 0.13) and these differences were strongly significant [β = 0.79, *t*(342) = 8.021, *p* < 0.001, *d* = 0.79]. In addition, both means were significantly different from the midpoint (4.00) of the scale (both *p* < 0.001).

### Socioeconomic Status and Nepotistic Behavioral Intentions

Participants who reported that they are higher on the McArthur measure reported stronger nepotistic behavioral intentions [β = 0.20, *t*(319) = 3.73, *p* < 0.001, *d* = 0.20].

### Situational Poverty and Nepotistic Behavioral Intentions

Participants belonging to the induced poor condition reported stronger nepotistic behavioral intentions (*M* = 4.56, *SE* = 0.14) than participants in the induced rich condition (*M* = 4.01, *SE* = 0.14). These differences were strongly significant [β = 0.30, *t*(342) = 2.78, *p* = 0.006, *d* = 0.30].

### Nepotistic vs. Non-nepotistic Behavioral Intentions

Participants who imagined themselves as someone doing a nepotistic act to support their family reported stronger behavioral intentions to act in the same manner (*M* = 4.69, *SE* = 0.14) than those participants who were presented the non-nepotistic scenario (*M* = 3.88, *SE* = 0.14); these differences were strongly significant [β = 0.43, *t*(342) = 4.13, *p* < 0.001, *d* = 0.43].

Some of the trends that we hypothesized came true: participants in imagined poverty situations were more accepting of nepotism (*M*_poor_ = 4.56; *M*_rich_ = 4.01), and participants who were richer in real life were more willing to engage in nepotism (continuous measure). However, we were incorrect in our judgment of cultures, where the participants from the more affluent United States had higher nepotistic intentions than those from Hungary (*M*_US_ = 5.00; *M*_HU_ = 3.53).

**FIGURE 1:**
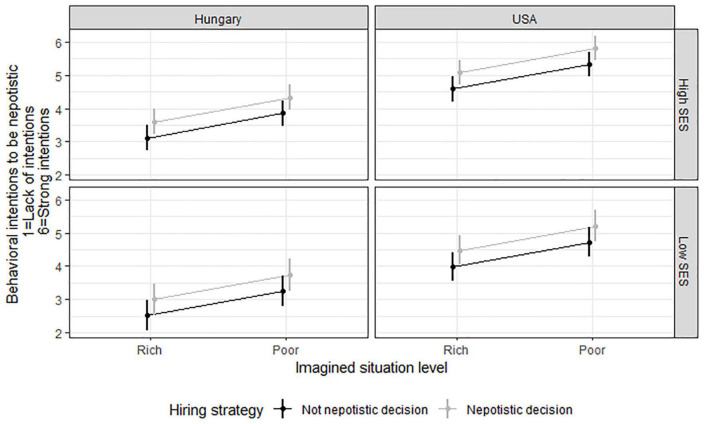
Differences in nepotistic behavioral intentions between cultures (United States vs. Hungary), self-reported low vs. high SES, imagined poverty vs. richness, and nepotistic vs. non-nepotistic hiring strategy. For the sake of clarity, “High” and “Low” SES within each culture was based on a median split of MacArthur ladder data (Med = 6).

If we inserted the control variables in separate regression models or if all of the predictors were present in the same OLS regression model, their main effect also remained strongly significant (see Figure 1).

### Cross-Cultural Level and Character Evaluation

U.S. participants reported more positive character evaluation (*M* = 3.39, *SE* = 0.07) than Hungarians (*M* = 2.81, *SE* = 0.07) and these differences were strongly significant [β = 0.66, *t*(340) = 6.43, *p* < 0.001, *d* = 0.66].

### Socioeconomic Status and Character Evaluation

Participants who reported that they are higher on the McArthur measure reported more favorable character evaluations [β = 0.16, *t*(318) = 2.92, *p* = 0.004, *d* = 0.16].

### Situational Poverty and Character Evaluation

Participants belonging to the induced poor condition reported more positive character evaluation (*M* = 3.34, *SE* = 0.07) than participants in the induced rich condition (*M* = 2.88, *SE* = 0.07). These differences were strongly significant [β = 0.51, *t*(340) = 4.91, *p* < 0.001, *d* = 0.51].

### Nepotistic vs. Non-nepotistic Hiring Decision and Character Evaluation

Participants who evaluated someone doing a nepotistic act reported more positive character evaluation (*M* = 3.37, *SE* = 0.07) than those who did not engage in nepotism (*M* = 2.85, *SE* = 0.07), and these differences were strongly significant [β = 0.58, *t*(340) = 5.55, *p* < 0.001, *d* = 0.58].

**FIGURE 2:**
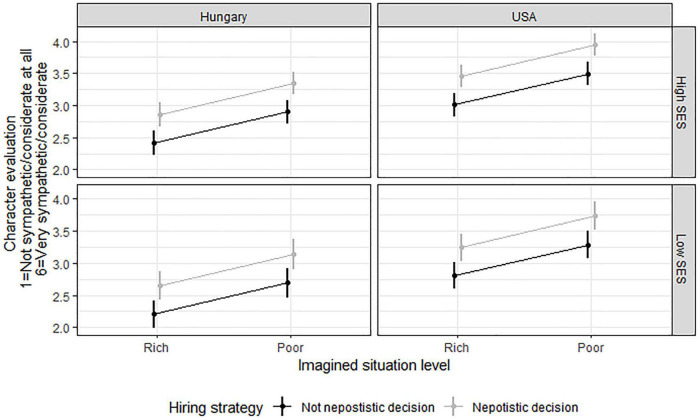
Positive Character Evaluation of a Nepotistic Person. For the sake of simplicity, “High” and “Low” SES within each culture was based on a median split of MacArthur ladder data (Med = 6). The cultural, SES, imagined situational and hiring strategy (nepotistic vs. non-nepotistic) differences are significant.

If all of the predictors were simultaneously present in the same regression analysis and or demographic variables were added (gender, age, level of education), their effect also remained strongly significant (see Figure 2).

## Discussion

Nepotism is not inherently good or bad. From the perspective of the employer, hiring a kin might lead to more trust between the two persons. The employer might express their genuine love and care toward the hired family member. Providing a livelihood can be seen as a benevolent and caring act from the perspective of the extended family. From the perspective of the employed person—especially in threatening economic circumstances—this act might provide a long-term sense of security. However, this is only one side of the coin. From an external point of view—more specifically from the perspective of the person who did not get the job based on their merits—demonstrates the other side of the coin.

A few months after the pandemic and biggest job loss crisis in US history, we found an almost a two standard deviation difference between low socioeconomic status Hungarians who imagined themselves rich in an online psychological experiment and rich US citizens who imagined themselves poor. These very preliminary results suggests that the social milieu including cultural, sociological and psychological forces (culture, individual social-class, high-level threats, and interpersonal relations) can simultaneously influence nepotistic intentions. These results might draw the attention to the relevance of a social-psychological explanation of nepotism.

In the present preliminary study, we analyzed three levels (cultural, socioeconomic, and imagined situational) in which poverty manifests itself. Most of the trends that we hypothesized came true: participants who were in imagined poverty situations were more accepting of nepotism, and participants who were richer in real life were more willing to engage in nepotism. However, we were incorrect in our predictions regarding cultural differences surprisingly participants from the affluent United States had higher nepotistic intentions than those from Hungary in our preliminary and non-representative study.

### Cultural Differences

We expected that similarly to other collaborative forms of illicit behaviors, such as corruption (Aidt, 2009) or academic cheating (Orosz et al., 2018), nepotistic behavioral intentions (Van de Vliert, 2011) would be more strongly present among less affluent Hungarian respondents than among more affluent US respondents based on prior cross-cultural studies and Eastern-European socio-historical background. In Hungary, family values became incredibly salient after WWII as extra-familial networks like unions, clubs, and other organizations disappeared (Hankiss, 1989), and familial ties became stronger, possibly providing ground or rationalizing the use of nepotism.

In the US context there are different value-backgrounds with regard to nepotism. In typically Western, individualistic cultures, people tend to characterize achievement in the job market based on their own individual morals or dreams (Spence, 1985). In this cultural context, nepotism is most viewed with a negative connotation (even being referred to as an “inherent evil”; Olsson, 2017), but it can also be seen as a positive factor by lowering unemployment. This side can become especially prevalent in high employment threat situations. For example, during the pandemic, corporate strategies shifted to mimic family businesses, specifically with regard to community-focused actions (Rivo-López et al., 2021). The preference for in-group favoritism might bring with it a reliance on practices like nepotism and it is likely that the usage of such tactics might have become more popular under COVID-19.

### Socioeconomic Level

There is a strong scientific consensus that people with higher SES are more likely to use unethical behaviors than their peers with lower SES (e.g., Piff et al., 2012). The present result strongly supports these prior scientific findings. However, it is beyond the scope of this preliminary study to identify the specific reasons behind these socio-economic differences. It might be related to greater independence, self-focused social cognition, selfishness, narcissistic tendencies or entitlement (Piff et al., 2010; Piff, 2014; Kraus et al., 2011, 2012; Piff et al., 2012). However, it is not guaranteed that this phenomena can be explained by a psychological mechanism that is related to pure self-interest in the case of nepotism. It might be possible that people with higher SES have a narrower sense of “self,” maintaining a self-interest that includes their family members, while people with lower SES might have a broader sense of “self”-interest including people who are less close to them. In this brief report we aimed to show these solid SES differences in imaginary nepotistic scenarios, and future studies might delve into the research on specific psychological mechanisms responsible for such differences.

### Imagined and Situational Level

At the imagined situational level, participants followed the trends we predicted: people who imagined that they were poor, reported stronger behavioral intentions to act nepotistically. These results align with the theory from Kahneman and Tversky (1979), as an imagined poor situation can be perceived as a threat. Therefore, people may sometimes take dangerous or morally unacceptable measures to reduce that risk in their lives. This loss aversion in threatening situations does not only induce risk-seeking behaviors, but also provides an appropriate rationalization of behaving unethically (Heinemann, 2014).

### Limitation

Naturally, this preliminary work is not without its limitations. First, as this was a preliminary study, we did not use a standardized approach of data collection, therefore the samples were not representative for Hungarian or US populations; therefore, the cross-cultural comparisons should be dealt with and require further in-depth investigations using representative samples. Second, instead of measuring actual hiring behavior in nepotistic situations, we implemented a self-reported vignette method that was evaluated with survey items. Future studies might consider using behavioral methods. Third, we used a single item measure of behavioral intentions; however, it appears that the character evaluation measure that included six items led to very similar results. Fourth, the demographic characteristics of the US and Hungarian respondents were very different; however, as we controlled for these differences the main results did not change in the case of both the behavioral intentions or the character evaluation. Fifth, the study was run in a very unusual period of time, when uncertainty was very strong in the US labor force; therefore, we suspect that cultural differences might be different if these questions are asked in a less unstable period of time. Sixth, besides poverty, there might be other relevant cultural differences between Hungary and the US. Seventh, future studies can identify differences between nepotistic and non-nepotistic decisions by contrasting more sharply the two options.

## Conclusion

The most important goal of the present preliminary work was to orient the scientific attention to the role of social psychology in nepotism as it appears to be an expression of care for one’s loved ones in a dangerous environment embedded in cultural, socioeconomic, and situated contexts. Our most important and somewhat paradoxical result is that nepotistic behavioral intentions were strong among affluent participants who found themselves in temporary financial threats. It appears that a combination of imagined situational poverty and high socioeconomic status may be responsible for nepotistic decisions. Besides, the present preliminary and non-representative data (as a small scale-scale “case study”) raises the question that, under threatening circumstances such as the greatest unemployment level (COVID-19) in the history of the United States, even US participants who are culturally the most against nepotism, can tend to consider nepotistic decisions. Further studies are needed to confirm these results and uncover the underlying psychological mechanisms.

## Data Availability Statement

The raw data supporting the conclusions of this article will be made available by the authors, without undue reservation.

## Ethics Statement

The studies involving human participants were reviewed and approved by the Eötvös Loránd University Faculty of Education and Psychology Institutional Review Board of Ref# 2020/368. The patients/participants provided their written informed consent to participate in this study.

## Author Contributions

LJ, ÉG, and GO contributed to the study design, literature review, data gathering, manuscript writing, data analyses and interpretation, and manuscript writing. All authors commented on the draft and contributed to the final version, approved the publication of the manuscript, and agreed to be accountable for all aspects of the work.

## Conflict of Interest

The authors declare that the research was conducted in the absence of any commercial or financial relationships that could be construed as a potential conflict of interest.

## Publisher’s Note

All claims expressed in this article are solely those of the authors and do not necessarily represent those of their affiliated organizations, or those of the publisher, the editors and the reviewers. Any product that may be evaluated in this article, or claim that may be made by its manufacturer, is not guaranteed or endorsed by the publisher.
