# Supplementary material for: Nepotistic Hiring and Poverty From Cultural, Social Class, and Situational Perspectives
Source: Front Psychol. 2022 Mar 22;13:780629. doi: 10.3389/fpsyg.2022.780629 (PMC8980472; doi:10.3389/fpsyg.2022.780629)
Supplement: Supplementary file 1 [file Data_Sheet_1.docx]

**Supplemental Materials**

**Table 1**

*Participants’ demographic characteristics*

|  | **Hungarian sample (**N = 191) | **US sample** (N = 171) | **tests** |
| --- | --- | --- | --- |
| **Gender** | 62% female  16.5% male  21.5% missing | 40.9% female  57.6% male  2.3% missing | X^2^(1, N=320)=47.18, p<0.001 |
| **Age** | M=23.91  SD=6.51 | M=33.77  SD=12.88 | t(318)=-8.49, p<0.001 |
| **Minority status** | 44% minority | 32.4% minority | X^2^(1, N=321)=15.86, p<0.001 |
| **SES** MacArthur | M = 6.27  SD = 1.34 | M = 6.44  SD = 2.03 | *t* = -0.912, *p* = 0.319 |

**OLS regression analysis results**

**Nepotistic behavioral intentions in the U.S. and in Hungary**

Call:

lm(formula = scale(nepo_rich_indep_beh) ~ culture, data = d)

Residuals:

    Min      1Q  Median      3Q     Max

-2.1577 -0.8235  0.0000  0.5394  1.8736

Coefficients:

             Estimate Std. Error t value Pr(>|t|)

(Intercept) -0.40432    0.07067  -5.721 2.31e-08 ***

cultureus    0.79478    0.09909   8.021 1.69e-14 ***

---

Signif. codes:

0 ‘***’ 0.001 ‘**’ 0.01 ‘*’ 0.05 ‘.’ 0.1 ‘ ’ 1

Residual standard error: 0.9188 on 342 degrees of freedom

  (23 observations deleted due to missingness)

Multiple R-squared:  0.1583, Adjusted R-squared:  0.1559

F-statistic: 64.34 on 1 and 342 DF,  p-value: 1.689e-14

 culture emmean     SE  df lower.CL upper.CL

 hun     -0.404 0.0707 342   -0.543   -0.265

 us       0.390 0.0695 342    0.254    0.527

Results are given on the scale(4.28, 1.85) (not the response) scale.

Confidence level used: 0.95

**Socioeconomic status and nepotistic behavioral intentions**

Call:

lm(formula = scale(nepo_rich_indep_beh) ~ scale(ladder), data = d)

Residuals:

    Min      1Q  Median      3Q     Max

-2.1098 -0.7972  0.1648  0.8210  1.8280

Coefficients:

              Estimate Std. Error t value Pr(>|t|)

(Intercept)    0.03420    0.05458   0.627  0.53137

scale(ladder)  0.20370    0.05467   3.726  0.00023 ***

---

Signif. codes:

0 ‘***’ 0.001 ‘**’ 0.01 ‘*’ 0.05 ‘.’ 0.1 ‘ ’ 1

Residual standard error: 0.9779 on 319 degrees of freedom

  (46 observations deleted due to missingness)

Multiple R-squared:  0.04171, Adjusted R-squared:  0.03871

F-statistic: 13.88 on 1 and 319 DF,  p-value: 0.0002298

**Socioeconomic status and nepotistic behavioral intentions**

Call:

lm(formula = scale(nepo_rich_indep_beh) ~ cond_nepo_rp, data = d)

Residuals:

    Min      1Q  Median      3Q     Max

-1.9183 -1.0819  0.2394  0.7788  1.6152

Coefficients:

              Estimate Std. Error t value Pr(>|t|)

(Intercept)   -0.14589    0.07486  -1.949  0.05214 .

cond_nepo_rp1  0.29695    0.10681   2.780  0.00573 **

---

Signif. codes:

0 ‘***’ 0.001 ‘**’ 0.01 ‘*’ 0.05 ‘.’ 0.1 ‘ ’ 1

Residual standard error: 0.9903 on 342 degrees of freedom

  (23 observations deleted due to missingness)

Multiple R-squared:  0.0221, Adjusted R-squared:  0.01924

F-statistic:  7.73 on 1 and 342 DF,  p-value: 0.005731

 cond_nepo_rp emmean     SE  df lower.CL upper.CL

 0            -0.146 0.0749 342 -0.29314  0.00136

 1             0.151 0.0762 342  0.00123  0.30091

Results are given on the scale(4.28, 1.85) (not the response) scale.

Confidence level used: 0.95

**Nepotistic vs. non-nepotistic behavioral intentions**

Call:

lm(formula = scale(nepo_rich_indep_beh) ~ cond_nepo_dep, data = d)

Residuals:

    Min      1Q  Median      3Q     Max

-1.9885 -1.0141  0.1692  0.7086  1.6830

Coefficients:

               Estimate Std. Error t value Pr(>|t|)

(Intercept)    -0.21370    0.07389  -2.892  0.00407**

cond_nepo_dep1  0.43499    0.10541   4.126 4.63e-05***

Signif. codes:

0 ‘***’ 0.001 ‘**’ 0.01 ‘*’ 0.05 ‘.’ 0.1 ‘ ’ 1

Residual standard error: 0.9774 on 342 degrees of freedom

  (23 observations deleted due to missingness)

Multiple R-squared:  0.04743, Adjusted R-squared:  0.04464

F-statistic: 17.03 on 1 and 342 DF,  p-value: 4.631e-05

 cond_nepo_dep emmean     SE  df lower.CL upper.CL

 0             -0.214 0.0739 342  -0.3590  -0.0684

 1              0.221 0.0752 342   0.0734   0.3692

Results are given on the scale(4.28, 1.85) (not the response) scale.

Confidence level used: 0.95

**Cross-cultural level and character evaluation**

Call:

lm(formula = scale(nepo_char) ~ culture, data = d)

Residuals:

     Min       1Q   Median       3Q      Max

-2.67552 -0.62603  0.03124  0.59019  2.82601

Coefficients:

            Estimate Std. Error t value Pr(>|t|)

(Intercept) -0.33632    0.07318  -4.596 6.09e-06 ***

cultureus    0.65727    0.10230   6.425 4.46e-10 ***

---

Signif. codes:  0 ‘***’ 0.001 ‘**’ 0.01 ‘*’ 0.05 ‘.’ 0.1 ‘ ’ 1

Residual standard error: 0.9457 on 340 degrees of freedom

  (25 observations deleted due to missingness)

Multiple R-squared:  0.1083, Adjusted R-squared:  0.1056

F-statistic: 41.28 on 1 and 340 DF,  p-value: 4.462e-10

 culture emmean     SE  df lower.CL upper.CL

 hun     -0.336 0.0732 340    -0.48   -0.192

 us       0.321 0.0715 340     0.18    0.462

Results are given on the scale(3.11, 0.895) (not the response) scale.

Confidence level used: 0.95

**Socioeconomic status and character evaluation**

Call:

lm(formula = scale(nepo_char) ~ scale(ladder), data = d)

Residuals:

     Min       1Q   Median       3Q      Max

-2.54339 -0.68055  0.06506  0.62401  2.20779

Coefficients:

               Estimate Std. Error t value Pr(>|t|)

(Intercept)    0.03630    0.05558   0.653  0.51420

scale(ladder)  0.16222    0.05559   2.918  0.00377 **

---

Signif. codes:

0 ‘***’ 0.001 ‘**’ 0.01 ‘*’ 0.05 ‘.’ 0.1 ‘ ’ 1

Residual standard error: 0.9943 on 318 degrees of freedom

  (47 observations deleted due to missingness)

Multiple R-squared:  0.02608, Adjusted R-squared:  0.02301

F-statistic: 8.515 on 1 and 318 DF,  p-value: 0.003774

**Situational poverty and character evaluation**

Call:

lm(formula = scale(nepo_char) ~ cond_nepo_rp, data = d)

Residuals:

   Min     1Q Median     3Q    Max

-2.617 -0.719  0.132  0.691  2.368

Coefficients:

               Estimate Std. Error t value Pr(>|t|)

(Intercept)   -0.25078    0.07316  -3.428 0.000683 ***

cond_nepo_rp1  0.51358    0.10469   4.906 1.45e-06 ***

---

Signif. codes:

0 ‘***’ 0.001 ‘**’ 0.01 ‘*’ 0.05 ‘.’ 0.1 ‘ ’ 1

Residual standard error: 0.9678 on 340 degrees of freedom

  (25 observations deleted due to missingness)

Multiple R-squared:  0.0661, Adjusted R-squared:  0.06335

F-statistic: 24.06 on 1 and 340 DF,  p-value: 1.447e-06

 cond_nepo_rp emmean     SE  df lower.CL upper.CL

 0            -0.251 0.0732 340   -0.395   -0.107

 1             0.263 0.0749 340    0.115    0.410

Results are given on the scale(3.11, 0.895) (not the response) scale.

Confidence level used: 0.95

**Nepotistic vs. non-nepotistic hiring decision and character evaluation**

Call:

lm(formula = scale(nepo_char) ~ cond_nepo_dep, data = d)

Residuals:

    Min      1Q  Median      3Q     Max

-2.6459 -0.7657  0.1488  0.7078  2.4017

Coefficients:

                Estimate Std. Error t value Pr(>|t|)

(Intercept)    -0.28462    0.07291  -3.904 0.000114***

cond_nepo_dep1  0.57597    0.10371   5.554 5.65e-08***

Signif. codes:

0 ‘***’ 0.001 ‘**’ 0.01 ‘*’ 0.05 ‘.’ 0.1 ‘ ’ 1

Residual standard error: 0.9589 on 340 degrees of freedom

  (25 observations deleted due to missingness)

Multiple R-squared:  0.08317, Adjusted R-squared:  0.08047

F-statistic: 30.84 on 1 and 340 DF,  p-value: 5.651e-08

 cond_nepo_dep emmean     SE  df lower.CL upper.CL

 0             -0.285 0.0729 340   -0.428   -0.141

 1              0.291 0.0738 340    0.146    0.436

Results are given on the scale(3.11, 0.895) (not the response) scale.

Confidence level used: 0.95
